# Increased RIPK4 expression is associated with progression and poor prognosis in cervical squamous cell carcinoma patients

**DOI:** 10.1038/srep11955

**Published:** 2015-07-07

**Authors:** De-Qing Liu, Fang-Fang Li, Jiang-Bo Zhang, Tie-Jun Zhou, Wen-Qiong Xue, Xiao-Hui Zheng, Yuan-Bin Chen, Xiao-Yu Liao, Lan Zhang, Shao-Dan Zhang, Ye-Zhu Hu, Wei-Hua Jia

**Affiliations:** 1 Sun Yat-Sen University Cancer Center; State Key Laboratory of Oncology in South China; Collaborative Innovation Center for Cancer Medicine, Guangzhou 510060, China; 2 Department of Pathology, The Affiliated Hospital of Luzhou Medical College, 319 Zhongshan Road, Luzhou, 646000, China

## Abstract

Aberrant expression of receptor interacting protein kinase 4 (RIPK4), a crucial regulatory protein of Wnt/β-catenin signaling, has recently been reported to be involved in several cancers. Here, we report the potential clinical implication and biological functions of RIPK4 in cervical squamous cell carcinoma (CSCC). One hundred and ninety-eight CSCC cases, 109 low-grade squamous intraepithelial lesions (LSILs), 141 high-grade squamous intraepithelial lesions (HSILs) and 63 chronic cervicitis were collected. The expression of RIPK4 was detected by immunohistochemistry (IHC), and its clinical value and oncogenic functions were further assessed. RIPK4 expression increased significantly with disease progression from 3.2% in chronic cervicitis, 19.3% in LSILs and 85.1% in HSILs to 94.4% in CSCCs (*P* < 0.001). Moreover, RIPK4 may serve as a useful biomarker to distinguish HSIL from chronic cervicitis/LSIL, which are two different clinical types for therapeutic procedures, with a high sensitivity and specificity (85.1% and 86.6%, respectively) and the performance improved when combined with p16^INK4a^. Further, RIPK4 overexpression was associated with overall (HR = 2.085, *P* = 0.038) and disease-free survival (HR = 1.742, *P* = 0.037). Knockdown of RIPK4 reduced cell migration and invasion via inhibition of Vimentin, MMP2 and Fibronectin expression in cervical cancer cells. RIPK4 might act as a potential diagnostic and independent prognostic biomarker for CSCC patients.

Cervical cancer is one of the most common gynecological cancers with 530,000 new cases and 275,000 deaths per year worldwide^1^. Effective screening, diagnosis and treatment strategies of cervical cancer have dramatically decreased the morbidity and mortality of this malignancy in developed countries^2^.

Histologically, for normal cervical epithelium to progress to cervical squamous cell carcinoma (CSCC), a series of precancerous lesions, including low-grade squamous intraepithelial lesions (LSILs) and high-grade squamous intraepithelial lesions (HSILs)^3^, must occur. Some studies have placed the treatment threshold of squamous intraepithelial lesions (SILs) at HSIL^4^. Therefore, one main issue for the management of cervical cancer is the diagnosis of HSIL versus LSIL. Recently, histology, which is thought of as the gold standard for the assessment of SIL, has also been found to be limited by intra- and interobserver variability^5^. Although some biomarkers, such as p16^INK4a^ and Ki-67, may provide an objective basis to support the histologic diagnosis of HSIL, they have been shown to have limited specificity^6^,^7^,^8^. Thus, there is an important clinical need to identify more specific biomarkers for detecting HSIL. Another issue for the management of cervical cancer is treatment. Radical hysterectomy and/or post-operative chemotherapy and radiotherapy are the standard treatment strategies for early-stage cervical cancer (International Federation of Gynecologists and Obstetricians (FIGO) stage IA1-IIA). However, clinical outcomes following the treatment of these patients vary significantly^9^. Given the need for personalized treatment and evaluation of treatment outcomes, searching for specific molecular biomarkers of clinical progression that predicting patient prognosis in cervical cancer has become important in the field of translational medicine. Although some potential molecular markers have been proposed to predict cervical cancer survival, satisfactory results have not yet been obtained^10^,^11^,^12^. Therefore, the identification of novel, specific biomarkers for use as predictive indicators and therapeutic targets for cervical cancer is urgently needed.

Receptor-interacting protein kinase 4 (RIPK4), a 91.6 kDa protein, is a serine/threonine kinase that belongs to the RIP kinase family. RIPK4 activates NF-kB and is required for keratinocyte differentiation^13^. One *Ripk4* mutant promotes autosomal recessive Bartsocas-Papas syndrome, which manifests with severe face, skin and limb abnormalities^14^,^15^. Recently, RIPK4 was reported to regulate the Wnt signaling pathway by phosphorylating Dishevelled, a receptor protein of the pathway, and its function was similar to that of Wnt3a. Moreover, RIPK4 was significantly upregulated in several types of cancer, including ovary, colorectal and skin tumors, and elevated RIPK4 expression promoted ovarian cancer in a xenograft tumor model^16^. These data suggest that RIPK4 may be an oncogene involved in the pathogenesis of malignant diseases. However, the expression and clinical significance of RIPK4 have not yet been elucidated in cervical cancer.

This study aimed to address two primary goals as follows: (a) to investigate the expression of RIPK4 in various stages of CSCC progression and to identify its clinical utility in diagnostic and prognostic significance, particularly in distinguishing HSIL from chronic cervicitis/LSIL; and (b) to determine the oncogenic functions and molecular mechanism of RIPK4 in cervical cancer cell lines. Our results suggested that RIPK4 was a novel oncogene in CSCC that could be used as an additional diagnostic and prognostic marker and potential therapeutic target for cervical cancer patients.

## Results

### RIPK4 expression is significantly upregulated in CSCC

**Figure 1:**
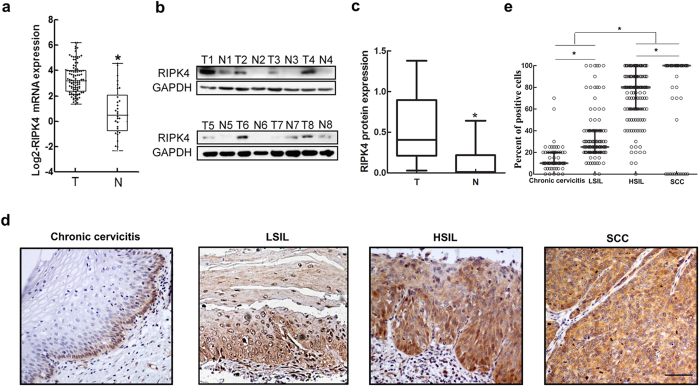
qRT-PCR, western blot and immunohistochemistry analyses of RIPK4. (**a**) qRT-PCR analysis of RIPK4 mRNA expression in 101 fresh CSCC tissues (T) and 30 paracancerous tissues (N). Expression levels were normalized to those of EFF1A1. (**b**) RIPK4 protein levels were determined by western blot in 8-paired CSCC tissues (T) and paracancerous tissues (N). GAPDH was used as a loading control. (**c**) Quantitative analysis of RIPK4 protein levels by western blot was presented as median ± interquartile. (**d**) RIPK4 immunohistochemical staining in representative cases from each cervical lesion. The scale bar represents 50 μm. (**e**) Expression of RIPK4 in each cervical lesion based on immunohistochemical scoring, as determined by the percentage of positive cells with strong staining. Values are the median with interquartile range.*P < 0.05 by the Wilcoxon rank-sum test.

The mRNA expression of the RIPK4 in 101 CSCC tissues was significantly higher than that of 30 paracancerous samples as determined by qRT-PCR (3.31 ± 1.19 *vs.* 0.50 ± 1.88, *P* < 0.001; Fig. 1a). Western blot analysis showed that RIPK4 protein expression was also significantly elevated in 7 of 8 CSCC cases compared with paired paracancerous tissues (*P* < 0.05; Fig. 1b,c).

### RIPK4 expression in chronic cervicitis, precancerous lesions and CSCC

We next evaluated the expression of RIPK4 protein and its subcellular localization in 198 cases of CSCC, 109 cases of LSIL, 141 cases of HSIL and 63 cases of chronic cervicitis using IHC. The clinical information of CSCC patients was summarized in Table 1. Notably, RIPK4 staining was mainly observed in the cytoplasm and rarely in the nucleus of epithelial cells. RIPK4 was consistently positive in the basal cell layer of almost all cervical squamous mucosa samples. RIPK4 staining was found in most HSIL and CSCC cases, but RIPK4 staining appeared less in LSILs and chronic cervicitis (Fig. 1d). RIPK4-positive cells in LSIL were mainly seen in the lower third epithelial layers (Supplementary Figure S1, row 2), and part of them extended beyond the lower third epithelial layers, even reaching to whole epithelial layers (Supplementary Figure S1, row 3).

Based on the cutoff value (≥50% of positive cells), positive RIPK4 expression was only observed in 2 cases of chronic cervicitis, and the rate of positive RIPK4 expression was 19.3%, 85.1% and 94.4% for LSIL, HSIL and CSCC, respectively, thereby demonstrating an increase with disease progression (Table 2, *P* < 0.001). The proportion of RIPK4-positive cells increased progressively from LSIL (25%) and HSIL (80%) to invasive CSCC (100%) (*P* < 0.001; Fig. 1e and Supplementary Table S1).

### RIPK4 as a biomarker for distinguishing HSIL from chronic cervicitis/LSIL

The progression of normal cervical epithelium to LSIL, HSIL and invasive cervical cancer is a continuous process, and current studies have placed the treatment threshold of SIL at HSIL^4^. Therefore, we analyzed RIPK4 and the traditionally reported biomarkers, p16^INK4a^ and Ki-67, in chronic cervicitis, LSIL and HSIL (Supplementary Figure S1 and Table 2). We further determined the diagnostic value (sensitivity, specificity and Youden’s index (YI)) of RIPK4, p16^INK4a^ and Ki-67 for distinguishing HISL from chronic cervicitis/LSIL. The results showed that RIPK4 had the highest diagnostic value (YI = 71.7) relative to p16^INK4a^ and Ki-67 (YI = 58.4 and YI = 52.4, respectively). The specificity of RIPK4 for distinguishing HSIL from chronic cervicitis/LSIL was higher than that of p16^INK4a^ (86.6% *vs.* 66.3%), but RIPK4 had slightly less sensitivity than p16^INK4a^ (85.1% *vs.* 92.1%). Ki-67 was inferior to RIPK4 and p16^INK4a^ for both sensitivity and specificity. More importantly, the results of combining two biomarkers with each other showed that the combination of RIPK4 and p16^INK4a^ had a higher YI diagnostic value of 73.5 with sensitivity of 79.1% and specificity of 94.4% compared to other combinations, RIPK4 alone or p16^INK4a^ alone (Table 2).

### Relationship between RIPK4 expression and clinicopathological characteristics of CSCC

**Figure 2:**
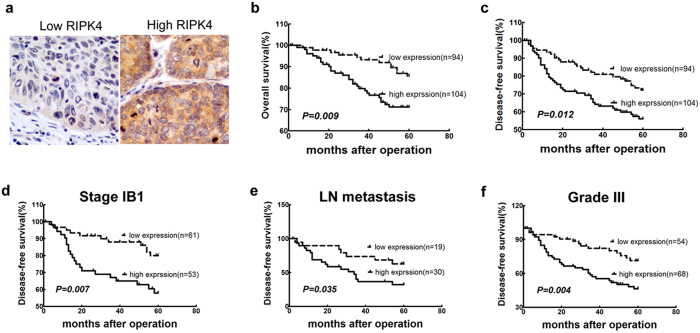
Survival curves for patients with CSCC according to high or low RIPK4 expression. (**a**) Immunohistochemical staining of RIPK4 in representative squamous cell carcinoma cases with low and high RIPK4 expression, respectively (magnification, 400×). (**b**) OS curves: 198 patients with low and high expression levels of RIPK4; (**c**) DFS curves: 198 patients with low and high expression levels of RIPK4. Disease-free survival analysis of RIPK4 expression in patients with (**d**) stage IB1, (**e**) LN metastasis or (**f**) histological grade III.

Based on the previously described cutoff value, RIPK4 expression in CSCC was separated into low and high groups (Fig. 2a). Low RIPK4 expression (IHC score<6.4) was observed in 47.5% (94 of 198) of CSCC samples, and high RIPK4 expression (≥6.4) was observed in 52.5% (104 of 198) of CSCC samples. The association between the expression of RIPK4 and the clinical significance of CSCC patients was summarized in Table 1. High RIPK4 expression was significantly related to clinical stage (60.7% *vs.* 46.5% for stage IB2-IIB and IB1; *P* = 0.048), tumor size (66.7% *vs.* 47.2% for > 4 cm and ≤4 cm; *P* = 0.017) and distant metastasis (72.0% *vs.* 49.7% for + and −; *P* = 0.037).

### Association among clinicopathological features, RIPK4 expression and CSCC patient survival

In total, 198 patients with CSCC were followed for up to 137 months with a median survival time of 60.5 months (1–137 months). The 5-year OS rate and disease-free survival (DFS) rate were 79.8% and 66.2%, respectively (Fig. 2b,c). Patients with high RIPK4 expression had a shorter 5-year OS than those with low RIPK4 levels (73.1% *vs.* 87.2%, *P* = 0.009; Fig. 2b). Similarly, DFS was significantly shorter in patients with high RIPK4 expression (Fig. 2c). Interestingly, individuals with RIPK4 overexpression had poorer DFS in several clinical stage subgroups as follows: IB1, lymph node (LN) metastasis and histological grade III (*P* = 0.007, *P* = 0.035 and *P* = 0.004, respectively; Fig. 2d,e,f). However, RIPK4 could not stratify patients in the following clinical stage subgroups: IB2-IIB, no LN metastasis, grade I/II, tumor size (≤4 cm) and tumor size (>4 cm) (*P* = 0.682, *P* = 0.169, *P* = 0.952, *P* = 0.084 and *P* = 0.222, respectively). Similar trends were observed for OS (data not shown). Univariate analysis using Cox’s proportional hazard model showed that RIPK4 expression, FIGO stages, histological grade, LN metastasis and tumor size were significantly related to OS and DFS, whereas age, HR HPV status and postoperative adjuvant therapy had no effect on OS and DFS (Supplementary Table S2). Multivariate Cox regression analysis indicated that RIPK4 expression, along with LN metastasis and histological grade, was significantly associated with OS (HR = 2.085 and *P* = 0.038 for RIPK4 expression; HR = 2.331 and *P* = 0.011 for LN metastasis; and HR = 2.199 and *P* = 0.040 for histological grade), and RIPK4 expression and LN metastasis were significantly associated with DFS (HR = 1.742 and *P* = 0.037 for RIPK4 expression; and HR = 1.987 and *P* = 0.010 for LN metastasis) in CSCC patients (Table 3).

### Effects of RIPK4 knockdown on CSCC cell growth, migration and invasion *in vitro*

**Figure 3:**
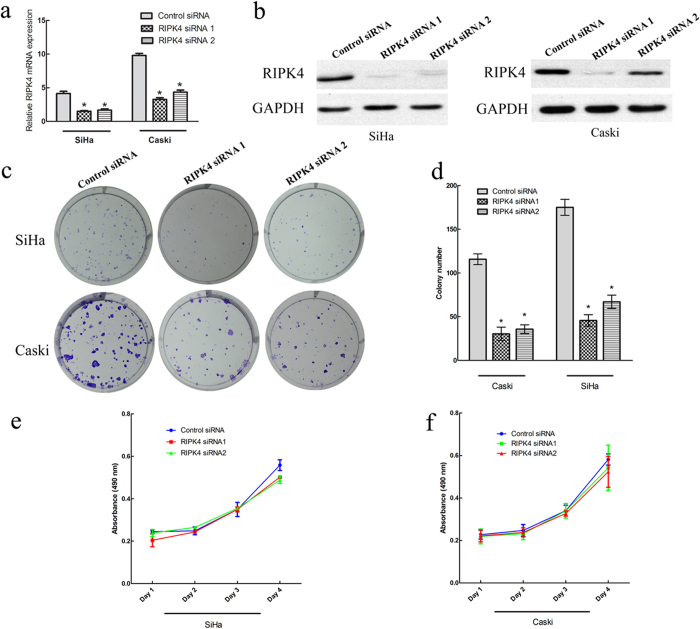
RIPK4 depletion decreases cell proliferation and colony formation capability in SiHa and Caski cells *in vitro*. (**a, b**) Real-time quantitative PCR analysis of RIPK4 mRNA expression and western blot analysis of RIPK4 protein expression in SiHa and Caski cells transfected with specific siRNAs targeting RIPK4 for 48 h. (**c, d**) Colony formation capacity of SiHa and Caski cells transfected with RIPK4 siRNAs and control siRNA were analyzed by colony formation assay. (**e**) Inhibition of SiHa cell proliferation by RIPK4 siRNAs tested by MTT assays. (**f**) Inhibition of Caski cell proliferation by RIPK4 siRNAs as measured by MTT assays. Values are the mean ± s.d. of three independent experiments. *P < 0.05 relative to the control.

**Figure 4:**
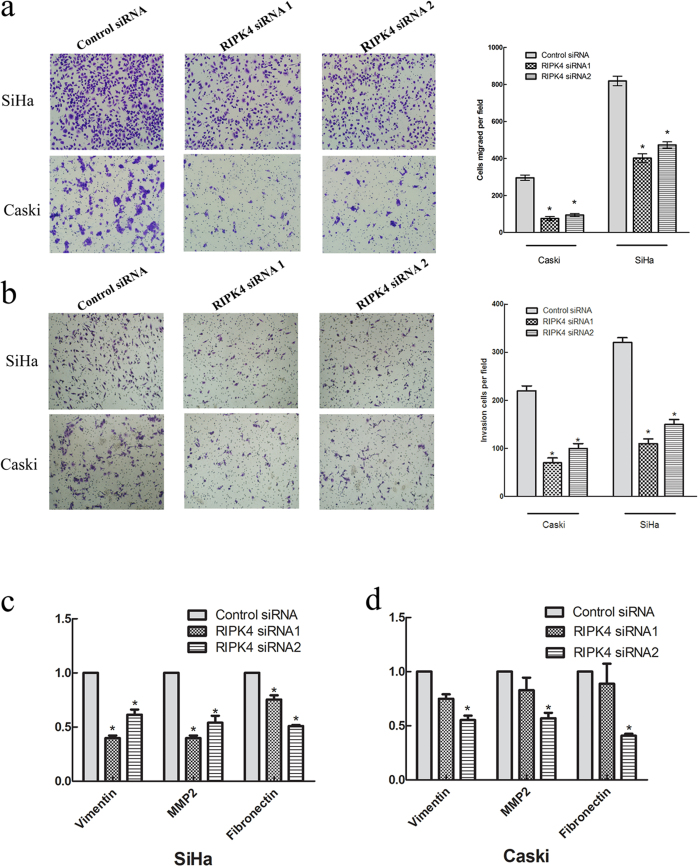
RIPK4 depletion decreases cell migration and invasion in SiHa and Caski cells *in vitro*. (**a**) Inhibition of SiHa and Caski cell migration by RIPK4 siRNAs as determined by migration assays. (**b**) Inhibition of SiHa and Caski cell invasion by RIPK4 siRNAs, as determined by Matrigel invasion assays. (**c, d**) RIPK4 was downregulated in SiHa and Caski cells, and the mRNA expression levels of Vimentin, MMP2 and Fibronectin were detected by qRT-PCR. GAPDH was used as an endogenous control. Values are the mean ± s.d. of three independent experiments. *P < 0.05 relative to the control.

To further investigate the functional role of RIPK4 in cervical cancer cells, we silenced its expression using two specific small interference RNAs (siRNAs) in SiHa and Caski cells. As shown in Fig. 3a,b, RIPK4 mRNA and protein levels were efficiently downregulated in cells transfected with specific siRNAs for RIPK4 compared with those transfected with control siRNA. We then assessed the impact of RIPK4 ablation on cell proliferation and clone formation *in vitro*. The cell proliferation and clone formation capacity were markedly inhibited after knockdown of RIPK4 expression in SiHa and Caski cells when compared with control siRNA treatment (*P* < 0.01; Fig. 3c,d,e,f). Collectively, these data suggested that RIPK4 played an important role in the growth of cervical cancer cells. In the migration assay, the cell migration rates were significantly suppressed after knockdown of RIPK4 expression in SiHa and Caski cells when compared with control siRNA treatment (*P* < 0.01; Fig. 4a). In addition, the Matrigel invasion assay demonstrated that knockdown of RIPK4 expression significantly suppressed the invasiveness of SiHa and Caski cells (*P* < 0.01; Fig. 4b).

### RIPK4 depletion downregulates Vimentin, MMP2 and Fibronectin expression in cervical cancer cells

A previous study has reported that RIPK4 promotes Wnt/β-catenin signaling activity in 293T cells^16^. Therefore, we first measured the activity of the Wnt/β-catenin pathway using the TOP/FOP-Flash reporter assay in 293T and SiHa cells. The results showed that RIPK4 overexpression significantly increased TOP-Flash activity in 293T cells (*P* < 0.05) but not in SiHa cells (Supplementary Figure S2).

To further explore the underlying mechanism of RIPK4 in promoting the migration and invasion of cervical cancer cells, we measured Vimentin, MMP2 and Fibronectin mRNA expression in RIPK4-siRNA-transfected cells and control cells. The results showed that Vimentin, MMP2 and Fibronectin mRNA expression levels were significantly inhibited after knockdown of RIPK4 expression in SiHa and Caski cells when compared with control siRNA treatment (*P* < 0.05; Fig. 4c,d).

## Discussion

To investigate if RIPK4 is a novel oncogene, we first assessed the expression of RIPK4 in several types of tumors by semi-quantitative PCR, and we found that RIPK4 was significantly upregulated in CSCCs (Supplementary Figure S3). Therefore, we then examined its expression in CSCC by qRT-PCR and western blot analysis, and we found that RIPK4 was significantly increased at both the mRNA and protein levels in most of the CSCC tissues. Consistent with these observations, IHC revealed that tumor tissues exhibited higher RIPK4 expression. Interestingly, we observed RIPK4 positive expression in most HSILs and SCC, whereas only 19.3% of the LSILs and 3.2% of chronic cervicitis were RIPK4 positive. To our knowledge, this is the first study to investigate the expression of RIPK4 and its potential clinical value in CSCC patients.

Some studies have reported that LSIL has a <1% risk of progression and is usually managed with Papanicolaou smear follow-up, whereas 5% to 20% of HSIL cases will progress to invasive carcinoma and are typically treated with colposcopy and an excisional procedure (loop electrosurgical excision procedure or cone biopsy)^17^. Therefore, accurate distinction of HSIL from LSIL is of great clinical significance for treatment recommendations. Histopathological assessment of SIL by pathologists is subjective^5^. Therefore, there is an important clinical need to identify specific biomarkers for HSIL. To compare the diagnostic efficiency of RIPK4 for HSIL with p16^INK4a^ and Ki-67, which are the most widely and consistently reported biomarkers^6^,^18^,^19^,^20^,^21^, we also conducted p16^INK4a^ and Ki-67 IHC staining. Keating *et al.* revealed that the staining of Ki-67 in the upper third of the epithelium being a strong indicator of HSIL and almost all HSILs were positive for Ki-67, while it was less reliable for LSIL, immature metaplasia and an inflammatory process, which can appear positive Ki-67 staining^22^. Some studies defined Ki-67 staining >1–2 layers of basal/parabasal and >50% epithelial cells as positivity^2^,^23^. So far, there was no consistent conclusion as to cutoff value of Ki-67 staining. In our study, in order to distinguish LSIL from HSIL better, Ki-67 staining was defined as positivity when there was continuous staining greater than the lower third of the epithelium, and we found that positive rate of Ki-67 was 80.7% in HSILs, similarly to the findings of Cavalcante *et al.* and Agoff *et al.* which defined the same cutoff value of Ki-67^24^,^25^.

We further analyzed the sensitivity, specificity and YI of RIPK4, p16^INK4a^ and Ki-67 for the diagnosis of HSIL versus chronic cervicitis/LSIL. The results revealed that RIPK4 was the optimal biomarker (YI = 71.7) for distinguishing HSIL from LSIL/chronic cervicitis relative to p16^INK4a^ (YI = 58.4) and Ki-67 (YI = 52.4). The sensitivity and specificity of p16^INK4a^ and Ki-67 for the diagnosis of HSIL in this study were similar to a previous report^23^. Van Niekerk *et al.* reported that combined staining of p16^INK4a^ and Ki-67 improves the specificities for the diagnosis of HSIL versus LSIL and chronic cervicitis^7^. In our study, we also assessed the diagnostic value of combining two biomarkers with each other, and we found that combined staining of RIPK4 and p16^INK4a^ had the highest diagnostic value (YI = 73.5) for distinguishing HSIL from LSIL and chronic cervicitis compared to other combinations, RIPK4 alone and p16^INK4a^ alone. To date, p16^INK4a^ remains the most reliable and sensitive ancillary marker, but it has a limited specificity for HSIL^2^,^26^. Therefore, a significant subset of cases diagnosed with HSIL would be excessively treated if staining of p16^INK4a^ alone was used. For this reason, RIPK4, especially the combination of RIPK4 and p16^INK4a^, with increased specificity for HSIL could help to more accurately diagnose, focus medical resources and avoid overtreatment of patients with cervical SIL.

In addition to its utility as a diagnostic marker for HSILs, we also revealed that high RIPK4 expression was associated with invasive and metastatic characteristics of CSCC, including advanced FIGO stage, larger tumor size and distant metastasis. These data indicated that increased RIPK4 expression may play a role in CSCC development and progression. More importantly, high RIPK4 expression was associated with the poor survival in CSCC patients. To our knowledge, this is the first report of the predictive value of RIPK4 expression in cancer prognosis. In this study, we demonstrated that increased RIPK4 expression was a strong and independent predictor of OS and DFS. Furthermore, high RIPK4 expression also indicated poorer DFS in patients who were stratified into the following subgroups: FIGO stage IB1, histological grade III and positive LN metastasis. Thus, RIPK4 expression has the potential to predict CSCC patient outcome. In addition, using an online survival analysis tool, we assessed the relationship between RIPK4 expression and survival of other types of cancer (http://kmplot.com/analysis/). Increased RIPK4 expression was found to be associated with lower overall survival in breast cancer and ovarian serous carcinoma (Supplementary Figure S4). Prognostic biomarkers that can predict clinical outcome, including treatment response and overall survival, have an important influence on the clinical management of CSCC patients. In this study, we demonstrated that RIPK4 was associated with the initiation and progression of CSCC, and we also showed that it could act as an independent prognostic marker. The diagnostic and prognostic value of RIPK4 for CSCC highlighted the potential clinical practicability of this new biomarker. Therefore, assessment of RIPK4 expression may serve as an additional tool for identifying patients who are at risk for tumor invasion or progression, and it may be a helpful predictor for proper management of individual therapy.

RIPK4, which contains an N-terminal RIP-like kinase domain and a C-terminal region characterized by the presence of 11 ankyrin repeats, belongs to a Ser/Thr kinase family that regulates signal transduction^27^. To date, few studies have investigated the role of RIPK4 in the pathogenesis of malignant diseases. Recently, Huang *et al.* reported that phosphorylation of Dishevelled by RIPK4 regulates the Wnt signaling pathway, and elevated RIPK4 expression promotes ovarian cancer in a xenograft tumor model^16^, thereby suggesting that RIPK4 may act as an oncogene. Increased RIPK4 mRNA levels have been reported in ovarian, skin and colorectal cancers^16^. However, RIPK4 expression is also reportedly downregulated in hepatocellular carcinoma and tongue squamous cell cancer, and it gradually decreases with the loss of differentiation from highly to poorly differentiated tongue squamous cell cancer^28^,^29^, thus implying that RIPK4 may act as a tumor suppressor. Additionally, RIPK4 expression has been shown to reduce the migration and invasion in Tca-8113 tongue cancer cells^29^. The discordance in these studies suggests that RIPK4 may have different carcinogenic mechanisms in different tumors.

To explore the possibility of using RIPK4 as a therapeutic target for CSCC, we used siRNA to knockdown endogenous RIPK4, and we analyzed the resulting phenotype in CSCC cells. Ablation of RIPK4 significantly inhibited cell proliferation of cervical cancer cells *in vitro*. This result was in accordance with the previously reported function of RIPK4 in ovarian cancer cell lines^16^. In addition, suppression of RIPK4 expression in cervical cancer cells significantly reduced cell migration and invasion *in vitro*, and high RIPK4 expression was positively correlated with distant metastasis in clinical samples, thereby suggesting that RIPK4 plays an important role in cervical cancer metastasis.

Tumor invasion and metastasis usually involve the abnormal activation of epithelial-mesenchymal transition (EMT), and this process can be regulated by multiple cellular signaling pathways, such as the Wnt/β-catenin signaling pathway. Previous studies have reported that RIPK4 regulates Wnt/β-catenin signaling activity in 293T cells^16^. Our results also confirmed that RIPK4 increased the transcription activity of β-catenin in 293T cells but not in CSCC cells, thereby indicating that the cancer-related functions of RIPK4 in CSCCs may not be explained by its regulating effects on Wnt/β-catenin signaling activity. Further studies revealed that suppression of RIPK4 expression significantly inhibited Vimentin, MMP2 and Fibronectin expression in two cervical cancer cell lines. Given that Vimentin, MMP2 and Fibronectin are well-known key players of the epithelial-mesenchymal transition (EMT) process in promoting tumor metastasis; our results indicated a pro-metastasis function of RIPK4 by promoting the EMT process in the progression of CSCC. Although we did not fully elucidate the mechanism by which RIPK4 promotes tumor metastasis, the observation of a distinct association between RIPK4 and tumor metastasis may have therapeutic and prognostic implications.

High-risk (HR) HPV infection is known as the main risk factor for cervical cancer^30^. Previous studies have reported that approximately 100% of SCC, 95.5–97.3% of HSIL, 58.3–75.0% of LSIL and 10.2–11.5% of normal women were HR HPV-positive in two Chinese cross-sectional screening studies^31^,^32^. In our study, we detected HR HPV subtypes in 143 of 198 CSCC cases, and fresh tissues from these cases were obtained and analyzed. The results showed that 94.4% of SCC were infected with HR HPV, which was consistent with a previous report, and there was no association between RIPK4 expression and HR HPV infection in CSCC patients. One of the shortcomings of this study was the lack of HPV status in the chronic cervicitis and SIL samples. Therefore, the presence of HR HPV infection in chronic cervicitis and SILs with RIPK4-positive or RIPK4-negative staining requires further study in the future.

In the present study, we examined RIPK4 expression in a relatively large cohort of cervical cancer specimens, including chronic cervicitis specimens and those determined to be pre-malignant and malignant by IHC, and we demonstrated that the expression level of RIPK4 can serve as an effective diagnostic biomarker for the diagnosis of HSIL versus chronic cervicitis/LSIL as well as a prognostic factor for CSCC. Furthermore, the oncogenic function and molecular mechanism of RIPK4 were verified in cervical cancer cell lines. However, this study had limitations because we did not include patients with stage III or IV disease, and most of these patients received radiotherapy or chemotherapy rather than surgery. The molecular mechanism underlying RIPK4-induced proliferation and metastasis in cervical cancer cell lines warrants further study.

In conclusion, RIPK4 was not only overexpressed in CSCC but was also associated with disease progression from the precancerous stage of LSIL and HSIL to CSCC. Moreover, we showed that RIPK4 could improve diagnostic specificity for HSIL and could serve as an independent prognostic factor for resectable CSCC patients. We also demonstrated that depletion of RIPK4 in cervical cancer cell lines using siRNA inhibited cell proliferation, migration and invasion capacity *in vitro*. These results strongly suggested that RIPK4 plays an important role in cervical carcinogenesis and that it is an additional diagnostic and independent prognostic marker. Furthermore, these results suggested that RIPK4 may be a potential therapeutic target for CSCC.

## Materials and Methods

### Patients and tissue specimens

In this study, 511 formalin fixed, paraffin embedded samples were retrospectively selected from the tumor biobank of Sun Yat-Sen University and Department of Pathology at the Affiliated Hospital of Luzhou Medical College between December 2001 and May 2009 (198 CSCC cases of stage FIGO I-II, 109 cases of low-grade squamous intraepithelial lesions (LSIL), 141 cases of high-grade squamous intraepithelial lesions (HSIL) and 63 cases of chronic cervicitis). Hematoxylin and eosin (H&E)-stained biopsies were reviewed, and pathologic diagnosis was confirmed. All cervical biopsies were independently classified by two to three expert pathologists as chronic cervicitis, LSIL and HSIL according to a majority diagnosis (≥2 of 3 agreeing). In total, 198 CSCC patients were followed for up to 137 months, with a median survival time of 60.5 months (1–137 months), by consulting follow-up medical records or calling the patients directly. The clinical information of the patients was summarized in Table 1. The criteria for case selection were as follows: (1) pathological diagnosis of primary squamous cell carcinoma; (2) no history of preoperative adjuvant therapy; and (3) no other history of cancer. In addition, 101 fresh CSCC tissues and 30 adjacent paracancerous samples were collected between January 2003 and January 2008 to detect the expression level of RIPK4. This study was approved by the medical ethics committee of Sun Yat-Sen University Cancer Center and informed consent was obtained from all subjects. All experimental methods were performed in accordance with approved guidelines of Sun Yat-Sen University Cancer Center.

### Tissue microarray (TMA) construction

Tissue microarray (TMA) blocks with 198 CSCC samples were generated according to the method described previously^33^. Briefly, hematoxylin and eosin (H&E)-stained sections were re-examined by an experienced pathologist to define and mark representative tumor regions. Cylindrical tissue specimens (1 mm diameter) were punched from representative tumor regions of the donor block and arrayed on a recipient paraffin block at a defined position. Two core samples from each case were also included using a tissue array instrument (ALPHELYS Minicore Instruments, France). The information for each block was recorded in a TMA map (spreadsheet), which indicated the position of each core. In total, 118 dots were included on each recipient paraffin block.

### Quantitative real-time polymerase chain reaction (qRT-PCR) analysis

Total RNA was extracted using TRIzol reagent (Invitrogen). Complementary DNA was synthesized using the PrimeScript RT reagent Kit (TaKaRa), according to the manufacturer’s instructions. qRT-PCR was performed using SYBR Green PCR master mix (Applied Biosystems) in a total volume of 10 μl using the Light-Cycler 480 instrument (Roche Diagnostics, Penzberg, Germany) with the following conditions: 95 °C for 5 min, followed by 45 cycles of 95 °C for 30 sec, 55 °C for 30 sec and 72 °C for 15 sec, and a final extension step of 72 °C for 5 min. The qRT-PCR results were calculated according to 2^−ΔΔCt^, and all experiments were repeated in triplicate. Primer sequences for gene expression detection are summarized in Supplementary Table S3.

### Western blot analysis

Total protein was extracted using 500 μl of RIPA buffer containing 1% protease inhibitor cocktail (Calbiochem). Tissue lysates were centrifuged at 15,000 g for 15 min at 4 °C, and protein concentrations were measured using the BCA protein assay kit (Pierce Chemical Company, Rockford, IL, USA). Total protein (40 μg) was separated by sodium dodecyl sulfate polyacrylamide gel electrophoresis and transferred to a polyvinylidene difluoride membrane (Millipore, Bedford, Mass, USA). A rabbit antibody against RIPK4 (1:500 dilution; Abgent, San Diego, USA) was used to detect RIPK4 protein, and a rabbit antibody against GAPDH protein (1:3000 dilution; Sigma, St. Louis, Mo., USA) was used as an internal control. Specific proteins were detected using an enhanced chemiluminescence kit (Pierce Chemical Company) according to the manufacturer’s instructions.

### HPV-DNA detection and typing

Genomic DNA was extracted from fresh tissue biopsies using the QIAamp DNA mini kit (QIAGEN, Hiden, Germany), according to the manufacturer’s instructions. The quality of the DNA was verified using a 102 bp sequence of β-globin to exclude false positive and false negative results.

HPV-DNA was detected using two-tube nested PCR. The first PCR (using MY09/11 primer) assay was performed using Taq PCR master mix (Applied Biosystems) in a total volume of 10 μl on a thermal cycler (Bio-Rad, California, USA) under the following conditions: 93 °C for 3 min, followed by 35 cycles of 95 °C for 30 sec, 53 °C for 30 sec and 72 °C for 30 sec, and a final extension step of 72 °C for 5 min. The nested-PCR (using GP5 + /GP6 + primer) master mix was used as described above, except that 2 μl of the diluted first nested-PCR was added as a template, and the amplification profile was as follows: denaturation of the template DNA for 1 cycle of 95 °C for 3 min; amplification of the target DNA for 35 cycles of 95 °C for 30 s, 43 °C for 30 s and 72 °C for 30 s; and a final extension for 5 min at 72 °C. The amplified DNA was verified by 1% agarose gel electrophoresis. Positive controls and negative controls (water) were included in each run for quality control.

HPV-positive samples were sent to Life Technologies for cycle-sequencing. Sequences were submitted to the NCBI nucleotide–nucleotide BLAST (blastn) website (http://www.ncbi.nlm.nih.gov/BLAST/) for specific genotyping.

### Cell culture and transfection

Cervical cancer cell lines (SiHa, Caski) were a kind gift from Dr. L.Z., and they were cultured in RPMI-1640 medium containing 100 U/ml penicillin, 100 μg/ml streptomycin and 10% FBS at 37 °C under 5% CO_2_.

Small interfering RNAs (siRNAs) targeting RIPK4 and negative control siRNA (control siRNA) were synthesized by Guangzhou RiboBio Co., Ltd. (Guangzhou, China). Cells were transfected with either RIPK4 or control siRNA using the Oligofectamine reagent (Invitrogen) according to the manufacturer’s instructions. The mRNA and protein levels were detected 48 h after transfection.

### MTT assay

Cells were plated into 96-well plates containing approximately 2 × 10^3^ cells per well 24 h after transfection. After 6 h, 20 μl of the 5 mg/ml MTT solution was added to each well and incubated for 4 h at 37 °C. The medium was then removed from each well, and 150 μl of DMSO was added to each well to solubilize the resultant MTT formazan. The absorbance was measured at 490 nm using a microplate reader (Bio-Rad). The experiment was repeated three times, and each experiment used six replicate wells.

### Colony formation assay

Cells were transfected with RIPK4 or control siRNA for 48 h and plated in a 6-well plate containing 1 × 10^3^ cells per well in triplicate. After 14 days, the plates were stained with crystal violet, and colonies containing more than 450 cells were counted using an Olympus phase-contrast microscope.

### Transwell migration assay and Matrigel invasion assay

The cell migration and invasion assays were both performed using a 24-well Transwell chamber with a pore size of 8 μm (Costar, New York, NY, USA). The inserts were coated with 50 μl of Matrigel (dilution of 1:2; BD Bioscience, Franklin Lakes, NJ, USA) for the Matrigel invasion assay. Cells (100,000) were plated into the upper chamber containing 100 μl of serum-free medium, and the lower chamber was filled with medium containing 20% FBS as a chemoattractant. After incubation for 24 h at 37 °C under 5% CO_2_ in a humidified incubator, the cells in the upper chambers were removed with a cotton tip, and the cells that migrated to the lower surface of the filter were fixed in 70% ethanol for 30 min and stained with 0.2% crystal violet for 10 min. Cell migration was scored by counting five random, high-power fields under a microscope. The experiment was repeated three times.

### Dual-luciferase reporter assay and plasmids

The pENTER and pENTER/RIPK4-overexpressing human RIPK4 plasmids were purchased from ViGene Biosciences (Shandong, China). For the luciferase reporter assay, 293 T and SiHa cells (1.5 × 10^5^) seeded in 24-well plates were transiently co-transfected with the TOP/FOP-Flash reporter and pTK-RL plasmids using Lipofectamine 2000 (Invitrogen, Carlsbad, CA, USA). At 24 h post-transfection, luciferase activity was analyzed using the Dual Luciferase Assay kit (Promega, Madison, WI, USA) according to the manufacturer’s instructions. Renilla was used as a co-reporter vector to normalize transfection efficiency. All experiments were performed three times with triplicate samples.

### Immunohistochemistry staining and assessment

Slides were dried at 60 °C for 2 h, deparaffinized for 10 min in xylene (twice), and rehydrated through a series of incubations in alcohol (100%, 100%, 95%, 90%, and 80%) for 5 min. The sections were soaked in 3% hydrogen peroxide for 10 min to block endogenous peroxidase activity and treated with ethylenediamine tetraacetic acid (EDTA) buffer (pH = 8.0) by pressure cooking for 7 min for antigen retrieval. Nonspecific reactivity was blocked with 10% normal goat serum for 30 min at room temperature. Subsequently, the slides were incubated with a rabbit polyclonal antibody against RIPK4 (1:150 dilution; Abgent, San Diego, USA), mouse monoclonal antibody against p16^INK4a^ (1:200 dilution; OriGene, USA) and mouse monoclonal antibody against Ki-67 (1:150 dilution; Abgent, San Diego, USA) overnight at 4 °C in a moist chamber and then incubated with anti-rabbit immunoglobulin antibody (ZSGB-BIO, Beijing, China) for 30 min at 37 °C. Finally, the slides were stained with DAB (3,3-diaminobenzidine) for 1 min, counterstained with Meyer’s hematoxylin (BASO Diagnostics, Inc. Zhuhai, China), dehydrated, and mounted. Known positive (breast cancer for RIPK4; and hepatocellular carcinoma for p16^INK4a^ and Ki-67) and negative (primary antibody with a normal rabbit IgG) controls were included.

Two independent investigators scored the immunoreactivity of all the cervical lesions slides randomly. For cervical biopsy samples, positive RIPK4 staining was defined as strong and continuous cytoplamic staining from basement membrane in at least half of the epithelial thickness (based on the fact that the basal cell layer is consistently positive in almost all cervical epithelial mucosa), those with no staining, focally positive, weakly staining or lacked the continuous staining were scored as negative. For p16^INK4a^, diffuse staining of nuclei with or without cytoplasmic reactivity (usually continuous staining from basement membrane in at least one third of the epithelial thickness) was scored as p16^INK4a^-positive as recently described by Darragh *et al.*^34^. No staining, rare single dispersed cell staining or noncontinuous stretches of epithelium were considered as p16^INK4a^ negative. The scoring of Ki-67 included nuclear staining only, and it was considered positive when there was continuous staining greater than the lower third of the epithelium. No staining, only cytoplasmic staining, 1–2 layers of basal/parabasal staining and/or scattered staining were scored as Ki-67 negative. Sensitivity, specificity and Youden’s index (YI) (YI = sensitivity + specificity-1), as a metric of accuracy, were calculated for the diagnoses of HSIL versus chronic cervicitis/LSIL. For invasive cervical cancer, the staining results of RIPK4 were scored using the following criteria: (i) percentage of positive tumor cells in the tumor tissue: 0 (0−10%), 1 (11%–25%), 2 (26%–50%), 3 (51%–75%) and 4 (76%–100%); and (ii) staining intensity: zero (no signal), 1 (weak), 2 (moderate) and 3 (strong). Final scores were calculated by multiplying the score for the percentage of positive cells by the intensity score (range 0–12). To display the relationship between RIPK4 expression and the clinicopathological characteristics and prognosis of CSCC, the best cut-off value for high and low levels of RIPK4 expression was determined using YI from ROC^35^. In our study, we inserted survival status into the YI to define the cutoff value. A high level of RIPK4 expression was defined as an average score ≥6.4, whereas low expression was defined as an average score <6.4.

### Statistical analysis

Statistical analysis was performed using the SPSS 16.0 software package (SPSS, Chicago, IL). The mRNA and protein expression of RIPK4 in fresh cancerous and para-cancerous tissues was evaluated using Wilcoxon rank-sum test. The chi-squared test was used to assess the correlation between RIPK4 expression and clinicopathological variables, including age, FIGO stage, tumor size, histological grade, lymph node metastasis, HPV status, local recurrence and distant metastasis, as well as the correlation between staining intensity of RIPK4, p16^INK4a^ and Ki-67 and the severity of cervical lesions. Overall survival (OS) was defined as the time from surgery to death. Disease-free survival (DFS) was defined as the time from surgery to local relapse or distant metastasis. Overall survival and disease-free survival curves were plotted using the Kaplan–Meier method and evaluated by the log-rank test. Univariate and multivariate survival analyses (adjusting for FIGO stage, histological grade, tumor size and LN metastasis) were performed using the Cox regression model. The results of the *in vitro* experiments were expressed as mean ± standard error from at least three separate experiments. Student’s t-tests were used to analyze differences between the two groups (RIPK4-siRNA group and control group). Differences were considered statistically significant when the 2-sided probability value was <0.05.

## Additional Information

**How to cite this article**: Liu, D.-Q. *et al.* Increased RIPK4 expression is associated with progression and poor prognosis in cervical squamous cell carcinoma patients. *Sci. Rep.* **5**, 11955; doi: 10.1038/srep11955 (2015).

## Supplementary Material

Supplementary Information

## Figures and Tables

**Table 1:** RIPK4 expression and clinicopathological variables in CSCC.

		RIPK4 expression level		
Clinicopathological variables	Number of cases	Low (%)	High (%)	*P*-value^a^
Age (years)				
≤40	82	38 (46.3)	44 (53.7)	0.788
**>**40	116	56 (48.3)	60 (51.7)	
FIGO stage				
IB1	114	61 (53.5)	53 (46.5)	**0.048**
IB2- IIB	84	33 (39.3)	51 (60.7)	
Tumor size				
≤4 cm	142	75 (52.8)	67 (47.2)	**0.017**
>4 cm	51	17 (33.3)	34 (66.7)	
Histological grade				
I/II	76	40 (52.6)	36 (47.4)	0.251
III	122	54 (44.3)	68 (55.7)	
Lymph node metastasis				
−	149	75 (50.3)	74 (49.7)	0.160
+	49	19 (38.8)	30 (61.2)	
High-risk HPV status				
−	8	4 (50.0)	4 (50.0)	0.951
+	135	66 (48.9)	69 (51.1)	
Local recurrence				
−	162	80 (49.4)	82 (50.6)	0.254
+	36	14 (38.9)	22 (61.1)	
Distant metastasis				
−	173	87 (50.3)	86 (49.7)	**0.037**
+	25	7 (28.0)	18 (72.0)	

Abbreviations: CSCC cervical squamous cell carcinoma.

^a^Chi-square test; FIGO International Federation of Gynecology and Obstetrics.

**Table 2:** Summary of immunohistochemistry data for cervical samples and clinical value of RIPK4, p16^INK4a^ and Ki-67 in relationship to diagnoses of HSIL.

	Chronic cervicitis	LSIL	HSIL	*P*^*a*^	HSIL *vs.* LSIL + chronic cervicitis		
Biomarkers	N (%)	N (%)	N (%)		Se	Sp	YI
RIPK4+	2/63 (3.2)	21/109 (19.3)	120/141 (85.1)	<0.001	85.1	86.6	**71.7**
p16^INK4a^+	8/59 (13.6)	48/107 (44.9)	128/139 (92.1)	<0.001	92.1	66.3	58.4
Ki-67+	9/60 (15.0)	38/106 (35.8)	113/140 (80.7)	<0.001	80.7	71.7	52.4
RIPK4+ and p16^INK4a^ +	0/57 (0)	9/103 (8.7)	110/139 (79.1)	<0.001	79.1	94.4	**73.5**
RIPK4+ or p16^INK4a^ +	10/57 (17.5)	56/103 (54.4)	137/139 (98.6)	<0.001	98.6	58.8	57.4
p16^INK4a^ + and Ki-67+	2/57 (3.5)	31/104 (29.8)	110/138 (79.7)	<0.001	79.7	79.5	59.2
p16^INK4a^ + or Ki-67+	13/57 (22.8)	51/104 (49.0)	130/138 (94.2)	<0.001	94.2	60.2	54.4
RIPK4+ and Ki-67+	0/59 (0)	6/103 (5.8)	102/140 (72.9)	<0.001	72.9	96.3	69.2
RIPK4+ or Ki-67+	10/59 (16.9)	51/103 (49.5)	129/140 (92.1)	<0.001	92.1	62.3	54.4

Abbreviations: LSIL low-grade squamous intraepithelial lesion; HSIL high-grade squamous intraepithelial lesion; Se sensitivity; Sp specificity; YI Youden’s index.

**Table 3:** Multivariate Cox regression analysis for survival in CSCC.

	OS		DFS	
Prognostic variables	Hazard ratio (95% CI)	*P*-value	Hazard ratio (95% CI)	*P*-value
RIPK4 expression	2.085 (1.040–4.182)	**0.038**	1.742 (1.035–2.932)	**0.037**
(High *vs.* Low)				
FIGO stage	1.255 (0.617–2.552)	0.531	1.155 (0.671–1.986)	0.603
(IB2- IIB *vs.*IB1)				
Histological grade	2.199 (1.038–4.658)	**0.040**	1.568 (0.914–2.692)	0.103
(III *vs.* I/II)				
Tumor size	1.642 (0.803–3.360)	0.174	1.331 (0.750–2.360)	0.329
(>4 cm *vs.* ≤4 cm)				
LN metastasis	2.331 (1.211–4.486)	**0.011**	1.987 (1.181–3.343)	**0.010**
(+ *vs.* −)				

Abbreviations: CSCC cervical squamous cell carcinoma; FIGO International Federation of Gynecology and Obstetrics; OS overall survival; DFS disease-free survival; CI confidence interval
