# Supplementary material for: Increased RIPK4 expression is associated with progression and poor prognosis in cervical squamous cell carcinoma patients
Source: Sci Rep. 2015 Jul 7;5:11955. doi: 10.1038/srep11955 (PMC4493702; doi:10.1038/srep11955)
Supplement: Supplementary Information [file srep11955-s1.doc]

**Supplementary information**

**Increased RIPK4 expression is associated with** **progression and poor prognosis in cervical squamous cell carcinoma patients**

De-Qing Liu^1#^, Fang-Fang Li^1#^, Jiang-Bo Zhang^1^, Tie-Jun Zhou^2^, Wen-Qiong Xue^1^, Xiao-Hui Zheng^1^, Yuan-Bin Chen^1^, Xiao-Yu Liao^1^, Lan Zhang^1^, Shao-Dan Zhang^1^, Ye-Zhu Hu^1^ and Wei-Hua Jia^1*^

^#^De-Qing Liu and Fang-Fang Li contributed equally to this work.

^*^Corresponding author: Wei-Hua Jia, PhD, Sun Yat-sen University Cancer Center; State Key Laboratory of Oncology in South China; Collaborative Innovation Center for Cancer Medicine, 651 Dongfeng East Road, Guangzhou 510060, China, Fax: (86-20) 8734 3392, e-mail: [jiaweih@mail.sysu.edu.cn](mailto:jiaweih@mail.sysu.edu.cn)

^1^Sun Yat-sen University Cancer Center; State Key Laboratory of Oncology in South China; Collaborative Innovation Center for Cancer Medicine, Guangzhou 510060, China

^2^Department of Pathology, The Affiliated Hospital of Luzhou Medical College, 319 Zhongshan Road, Luzhou, 646000, China

**Supplementary Table S1 Expression data of RIPK4 in each cervical lesion based on percentage of positively stained cells**

|  | Minimum | Maximum | P25 | P50 | P75 |
| --- | --- | --- | --- | --- | --- |
| Chronic cervicitis | 0^a^ | 70 | 10 | 10 | 20 |
| LSIL | 0 | 100 | 20 | 25 | 40 |
| HSIL | 0 | 100 | 60 | 80 | 100 |
| SCC | 0 | 100 | 100 | 100 | 100 |

Abbreviations: P25 lower quartile; P50 median; P75 upper quartile. ^a^percentage of positive stained cells

**Supplementary Table S2** Univariate analysis of different prognostic factors in 198 patients with CSCC

| Prognostic variables | OS | | DFS | |
| --- | --- | --- | --- | --- |
|  | HR (95% CI) | ***P*** | HR (95% CI) | ***P*** |
| RIPK4 level | 2.388 (1.214-4.698) | **0.012** | 1.868 (1.133-3.079) | **0.014** |
| (High *vs.* Low) |  |  |  |  |
| Age | 1.666 (0.847-3.277) | 0.139 | 1.254 (0.761-2.066) | 0.375 |
| (>40 *vs.* ≤40) |  |  |  |  |
| FIGO stage | 1.995 (1.066-3.735) | **0.031** | 1.660 (1.028-2.682) | **0.038** |
| (IB2-II*vs.*IB1) |  |  |  |  |
| Grade | 2.348 (1.118-4.933) | **0.024** | 1.736 (1.020-2.953) | **0.042** |
| (III *vs.* I/II) |  |  |  |  |
| Tumor size (cm) | 2.308 (1.219-4.373) | **0.010** | 1.743 (1.041-2.921) | **0.035** |
| (>4 *vs.* ≤4) |  |  |  |  |
| LN metastasis | 2.818 (1.511-5.257) | **0.001** | 2.290 (1.400-3.748) | **0.001** |
| (+ *vs.* −) |  |  |  |  |
| HR HPV infection | 22.285 (0.004-110373.484) | 0.475 | 1.031 (0.247-4.311) | 0.966 |
| (+ *vs.* −) |  |  |  |  |

Abbreviations: CSCC cervical squamous cell carcinoma; FIGO International Federation of Gynecology and Obstetrics; OS overall survival; DFS disease-free survival; CI confidence interval, HR HPV high-risk HPV, HR Hazard ratio.

**Supplementary Table S3 Primer sequences used in our study**

| Primers | Sequence (5’-3’) |
| --- | --- |
| RIPK4-F | CCATGTCCACTGGAAGACCT |
| RIPK4-R | GAGCCCGTCTCCATGTACTC |
| EFF1A1-F | CTGAGCGTGAACGTGGTATC |
| EFF1A1-R | ACACCAGCAGCAACAATCAG |
| Vimentin -F | ATCCAAGTTTGCTGACCTCTCTG |
| Vimentin -R | TTCCAGGGACTCATTGGTTCC |
| MMP2-F | ACAACTTCTTCCCTCGCAAG |
| MMP2-R | AGCGGCCAAAGTTGATCATG |
| Fibronectin-F | GCAAGCCCATAGCTGAGAAG |
| Fibronectin-R | TGGTAAACAGCTGCACGAAC |
| GAPDH-F | CTCCTCCTGTTCGACAGTCAGC |
| GAPDH-R | CCCAATACGACCAAATCCGTT |
| MY09 | CGTCCMARRGGAWACTGATC |
| MY11 | GCMCAGGGWCATAAYAATGG |
| GP5+ | TTTGTTACTGTGGTAGATACTAC |
| GP6+ | GAAAAATAAACTGTAAATCATATTC |
| β-globin-F | GTGCACGGACTCCTGAGGAGA |
| β-globin-R | CCTTGATACCAACCTGCCCAG |

**Supplementary Figures**

**
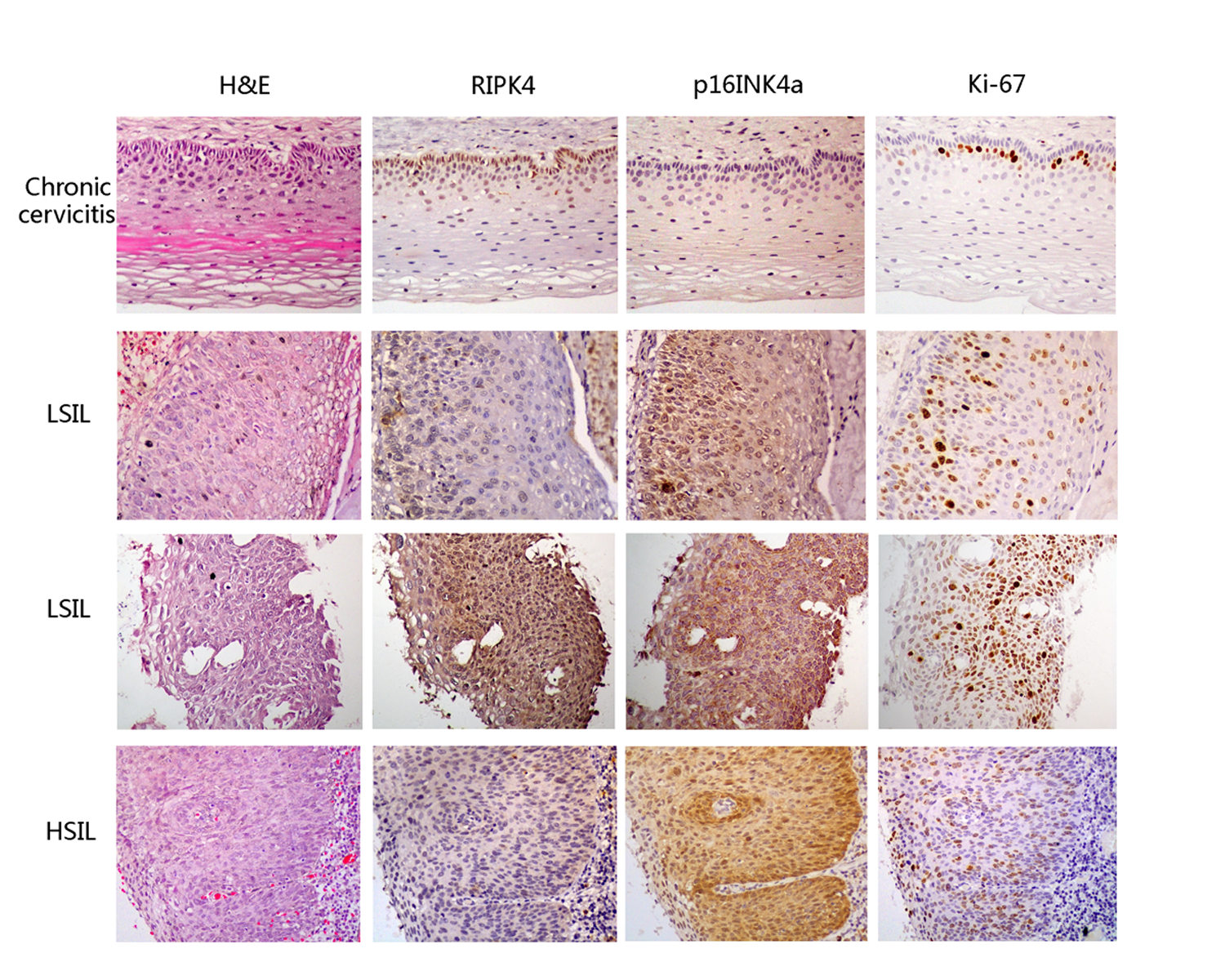
**

**Supplementary Figure S1. RIPK4, p16^INK4a^ and Ki-67 in different cervical lesions.** LSIL: low-grade squamous intraepithelial lesion; HSIL: high-grade squamous intraepithelial lesion. Row 1 is a chronic cervicitis that is negative for RIPK4, p16^INK4a^ and Ki-67. Row 2 is a case of LSIL that is negative for RIPK4 but positive for p16^INK4a^ and Ki67. Row 3 is a LSIL that is positive for RIPK4, p16^INK4a^ and Ki-67. Row 4 is an example of a HSIL that is positive for p16^INK4a^ and Ki-67 but negative for RIPK4. Images were taken at ×20 magnification.


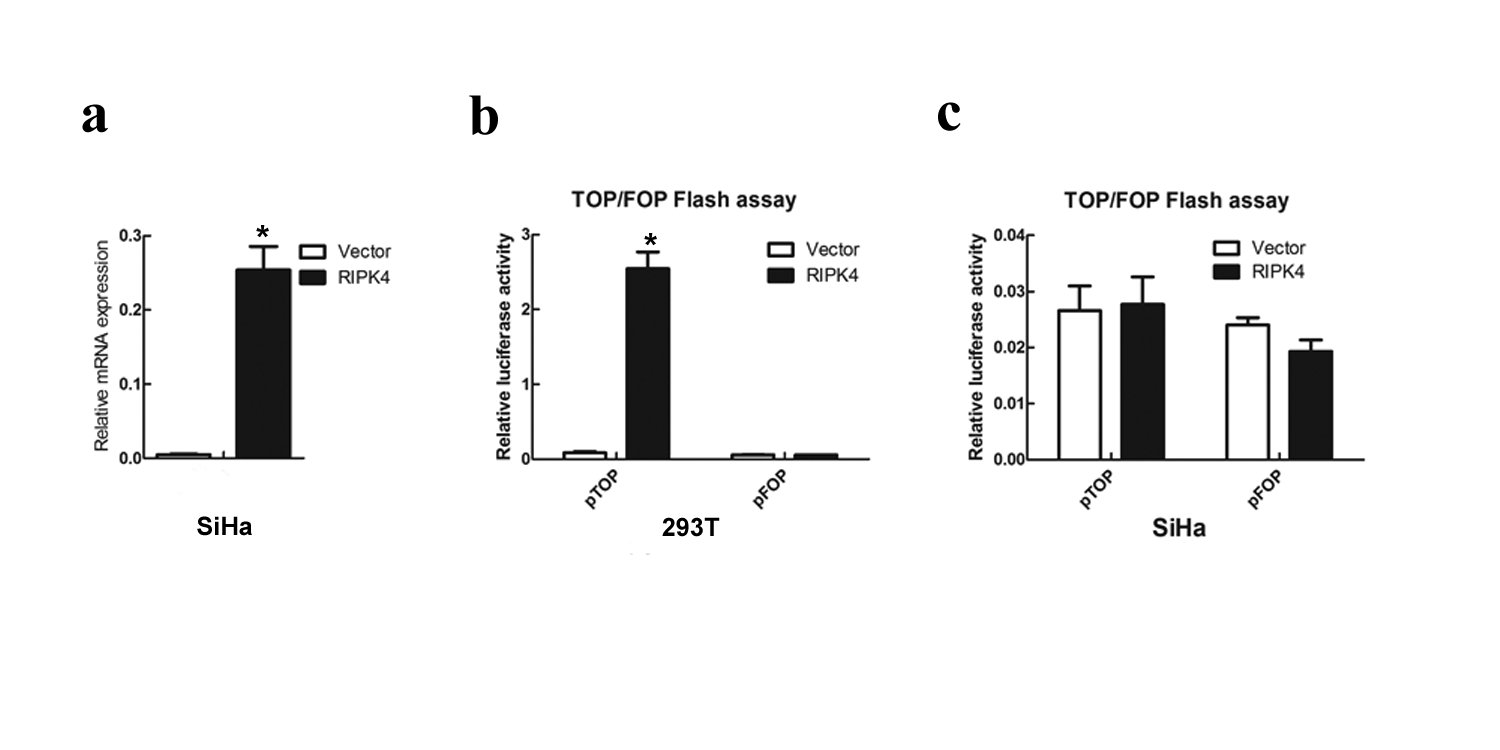


**Supplementary Figure S2.** **RIPK4 and Wnt/β-catenin pathway**.

RIPK4 mRNA expression in vector- and RIPK4-transduced SiHa cells (a). RIPK4-modulated 293T (b) and Siha (c) cells were transfected with the TOP/FOP-Flash reporter plasmid, and the reporter activities were detected 24 h after transfection by a luciferase assay. Values are the mean±s.d. of three independent experiments. **P*<0.05 relative to the control.


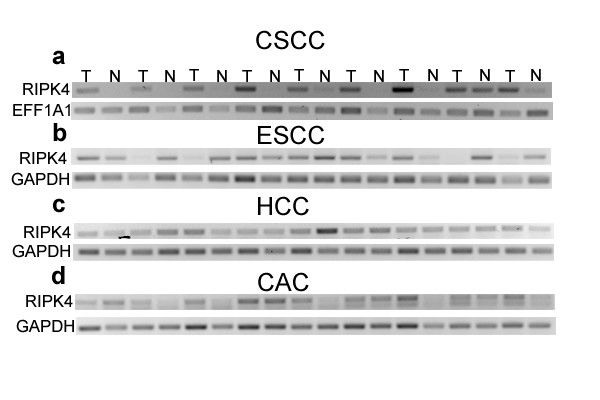


**Supplementary** **Figure S3. RIPK4 mRNA expression in CSCC, ESCC, HCC and CAC.** CSCC: Cervical squamous cell carcinoma, ESCC: Esophageal squamous cell carcinoma, HCC: Hepatocellular carcinoma, CAC: Colorectal adenocarcinoma. Semi-quantitative PCR analysis of RIPK4 mRNA expression in (a) 9 pairs CSCC tissues (T) and adjacent non-cancerous tissues (N); (b) 9 pairs ESCC T and N; (c) 9 pairs HCC T and N; (d) 9 pairs CAC T and N. Expression levels were normalized to EFF1A1 in CSCC, GAPDH in ESCC, HCC and CAC.


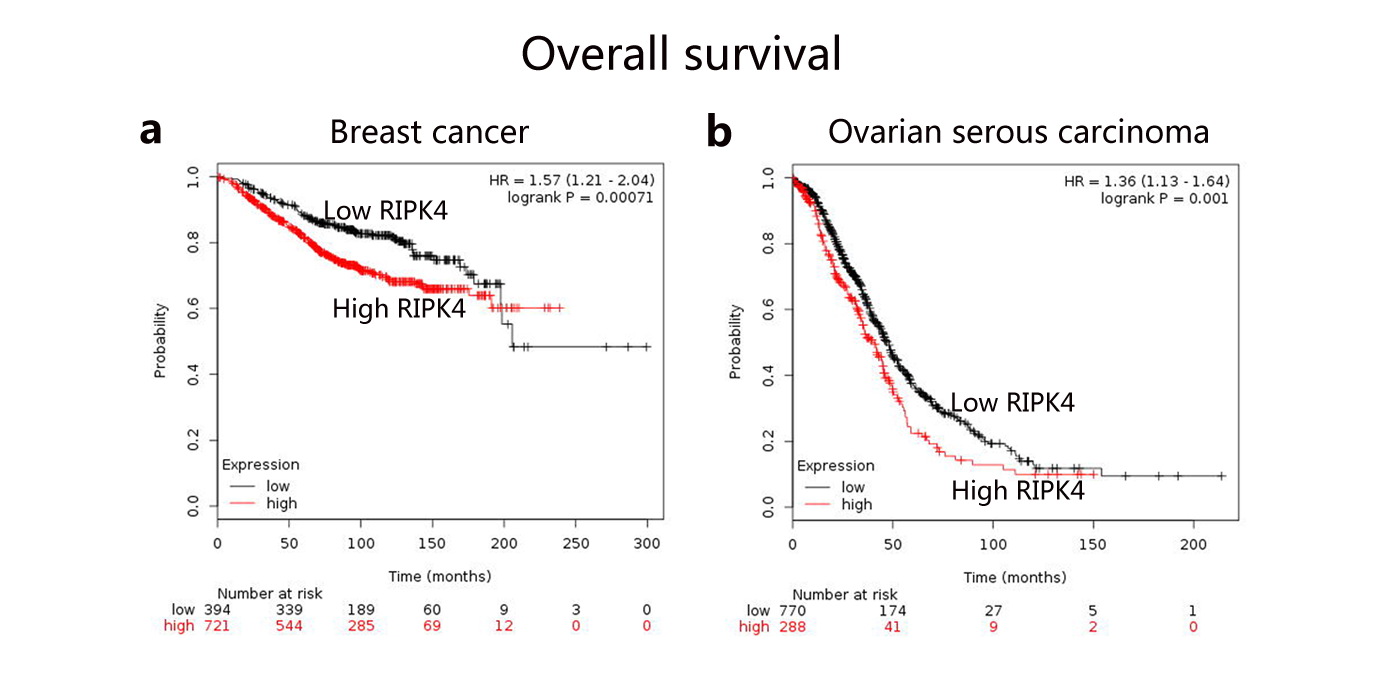


**Supplementary Figure S4.** **RIPK4 expression and overall survival in breast and ovarian cancers**. Kaplan-Meier plots of overall survival in patients with (a) breast cancer (b) ovarian serous carcinoma relative to RIPK4 expression.
